# Single-word comprehension deficits in the nonfluent variant of primary progressive aphasia

**DOI:** 10.1186/s13195-018-0393-8

**Published:** 2018-07-18

**Authors:** Jolien Schaeverbeke, Silvy Gabel, Karen Meersmans, Rose Bruffaerts, Antonietta Gabriella Liuzzi, Charlotte Evenepoel, Eva Dries, Karen Van Bouwel, Anne Sieben, Yolande Pijnenburg, Ronald Peeters, Guy Bormans, Koen Van Laere, Michel Koole, Patrick Dupont, Rik Vandenberghe

**Affiliations:** 1 Laboratory for Cognitive Neurology, Department of Neurosciences, KU Leuven, Herestraat 49, 3000 Leuven, Belgium; 2 Alzheimer Research Centre KU Leuven, Leuven Research Institute for Neuroscience & Disease, KU Leuven, Herestraat 49, 3000 Leuven, Belgium; 3 Neurology Department, University Hospitals Leuven, Herestraat 49 - box 7003, 3000 Leuven, Belgium; 4 Neurodegenerative Brain Diseases Group, Center for Molecular Neurology, VIB, Universiteitsplein 1, 2610 Antwerp, Belgium; 5 Institute Born-Bunge, Neuropathology and Laboratory of Neurochemistry and Behavior, University of Antwerp, Universiteitsplein 1, 2610 Antwerp, Belgium; 6 Neurology Department, University Hospitals Ghent, Corneel Heymanslaan 10, 9000 Ghent, Belgium; 7 Old Age Psychiatry Department, GGZinGeest, Van Hilligaertstraat 21, 1072 JX Amsterdam, The Netherlands; 8 Alzheimer Center & Department of Neurology, VU University Medical Center, De Boelelaan 1117, 1081 HV Amsterdam, The Netherlands; 9 Radiology Department, University Hospitals Leuven, Herestraat 49, Leuven, 30000 Belgium; 10 Laboratory of Radiopharmaceutical Research, KU Leuven, Herestraat 49, 3000 Leuven, Belgium; 11 Nuclear Medicine and Molecular Imaging, University Hospitals Leuven, Herestraat 49, 3000 Leuven, Belgium

**Keywords:** [^18^F]-THK5351, Mixed variant, Primary progressive aphasia, Frontotemporal dementia, Alzheimer’s disease, Tau, Positron emission tomography, Semantic, Amyloid

## Abstract

**Background:**

A subset of patients with the nonfluent variant of primary progressive aphasia (PPA) exhibit concomitant single-word comprehension problems, constituting a ‘mixed variant’ phenotype. This phenotype is rare and currently not fully characterized. The aim of this study was twofold: to assess the prevalence and nature of single-word comprehension problems in the nonfluent variant and to study multimodal imaging characteristics of atrophy, tau, and amyloid burden associated with this mixed phenotype.

**Methods:**

A consecutive memory-clinic recruited series of 20 PPA patients (12 nonfluent, five semantic, and three logopenic variants) were studied on neurolinguistic and neuropsychological domains relative to 64 cognitively intact healthy older control subjects. The neuroimaging battery included high-resolution volumetric magnetic resonance imaging processed with voxel-based morphometry, and positron emission tomography with the tau-tracer [^18^F]-THK5351 and amyloid-tracer [^11^C]-Pittsburgh Compound B.

**Results:**

Seven out of 12 subjects who had been classified a priori with nonfluent variant PPA showed deficits on conventional single-word comprehension tasks along with speech apraxia and agrammatism, corresponding to a mixed variant phenotype. These mixed variant cases included three females and four males, with a mean age at onset of 65 years (range 44–77 years). Object knowledge and object recognition were additionally affected, although less severely compared with the semantic variant. The mixed variant was characterized by a distributed atrophy pattern in frontal and temporoparietal regions. A more focal pattern of elevated [^18^F]-THK5351 binding was present in the supplementary motor area, the left premotor cortex, midbrain, and basal ganglia. This pattern was closely similar to that seen in pure nonfluent variant PPA. At the individual patient level, elevated [^18^F]-THK5351 binding in the supplementary motor area and premotor cortex was present in six out of seven mixed variant cases and in five and four of these cases, respectively, in the thalamus and midbrain. Amyloid biomarker positivity was present in two out of seven mixed variant cases, compared with none of the five pure nonfluent cases.

**Conclusions:**

A substantial proportion of PPA patients with speech apraxia and agrammatism also have single-word comprehension deficits. At the neurobiological level, the mixed variant shows a high degree of similarity with the pure nonfluent variant of PPA.

**Trial registration:**

EudraCT, 2014–002976-10. Registered on 13-01-2015.

**Electronic supplementary material:**

The online version of this article (10.1186/s13195-018-0393-8) contains supplementary material, which is available to authorized users.

## Background

Primary progressive aphasia (PPA) is a neurodegenerative syndrome characterized by an isolated language impairment with relative sparing of other cognitive domains [1]. Current consensus recommendations describe clinical criteria for three subtypes: a nonfluent/agrammatic variant (NFV), a semantic variant (SV), and a logopenic variant (LV) [1]. NFV PPA patients present with speech apraxia and/or agrammatism [1, 2], whereas SV PPA patients have single-word comprehension deficits and/or object recognition problems [1, 3, 4]. LV PPA patients experience word retrieval difficulties in spontaneous speech and are deficient on repetition tasks that have a high short-term phonological memory load [5, 6]. Even with the most careful clinical phenotyping, the link between the clinical phenotype and the underlying neuropathology remains probabilistic: 43–83% of NFV have underlying frontotemporal lobar degeneration (FTLD) tauopathy [7, 8] and 67%–88% of SV cases have FTLD transactive response DNA binding protein 43 kDa (TDP-43) type C pathology [7, 8]. Of the LV cases, 56–100% show underlying Alzheimer’s disease pathology [7–10].

The clinical diagnosis of NFV PPA is based on the presence of two core clinical features, namely speech apraxia and/or agrammatism, and at least two of the following three features: impaired comprehension of syntactically complex sentences indicative of agrammatism; spared single-word comprehension; or spared object knowledge [1]. Cases who have purely speech apraxia without clear agrammatism are sometimes classified as primary progressive apraxia of speech [11, 12], a disorder which has been set apart from PPA [12–14]. The phenotype of NFV PPA has been associated with loss of structural integrity of the dorsal language stream [15], implicated in speech production and grammatical processes [16, 17], while ventral language stream functions [18], such as single-word comprehension and object knowledge, remain relatively intact. However, a subset of NFV cases exhibit single-word comprehension deficits in addition to motor speech problems and/or agrammatism [7, 19, 20]. This has been proposed to constitute a fourth, ‘mixed’ variant (MV) of PPA [7, 19–21]. This variant is not formally recognized in the current diagnostic classification of PPA [1]. The current classification guidelines might be reconsidered since some studies report that 16–41% of PPA patients remain unclassified as they fit criteria for more than one PPA variant [20, 22–26]. Moreover, data-driven mathematical analyses of neurolinguistic and neuropsychological data of PPA patients confirm the existence of a separate, mixed variant [26]. Inclusion of a mixed phenotype into the list of variants raises the comprehensiveness of the classification from 80% to nearly 90% of PPA cases [20]. The mixed phenotype can emerge as a distinct clinical form in mild or early disease and is not merely due to a more advanced disease stage [20]. To date, limited neuroimaging data in small case series of MV PPA indicate left frontotemporal atrophy on structural magnetic resonance imaging (MRI) [20, 27]. In vivo amyloid positron emission tomography (PET) imaging in four MV cases showed amyloid-positivity in three out of four cases [10]. However, the distribution pattern of the other Alzheimer’s disease hallmark protein, i.e., tau, remains to be investigated on tau-PET. At pathological examination, in a series of six MV cases, four had underlying Alzheimer’s disease pathology [7] and, in another series, three out of four had FTLD tauopathy [27].

The presence of single-word comprehension problems together with speech apraxia or agrammatism is intriguing. Word comprehension relies on a distributed network [28]. The underlying mechanism and anatomical basis of the single-word comprehension problems may differ between PPA subtypes. In SV, word comprehension deficits have been related to damage to the anterior temporal cortex [29], while in LV the occurrence of word comprehension problems may be due to extension of damage into the posterior third of the superior temporal sulcus and into the middle temporal gyrus [30, 31]. Neuropsychological evidence of a role of inferior frontal and premotor cortex, the regions most prominently affected in NFV, in word comprehension is relatively scarce [32]. Functional imaging studies, however, in healthy subjects have revealed consistent evidence for the contribution of the pars triangularis [33], the inferior frontal sulcus, and the anterior inferior frontal gyrus [34] to the processing of word meaning. The left pars triangularis codes for the representation of the meaning of written and auditory words [33]. The dorsal part of the pars triangularis has also been implicated in semantic working memory [35], semantic selection [36], and semantic control [37]. Disintegration of the inferior frontal cortex in NFV may also alter predictive coding during speech perception [32]. The mechanisms for the word comprehension deficits in MV may therefore be fundamentally different from those underlying the word comprehension problems in SV or LV.

The primary objectives of this study were to assess the prevalence and nature of single-word comprehension problems in individual PPA patients with speech apraxia and/or agrammatism and to study their characteristics on multimodal imaging including high-resolution volumetric MRI, tau PET with [^18^F]-THK5351 [38], and amyloid PET with [^11^C]-Pittsburgh Compound B ([^11^C]-PIB) [39]. [^18^F]-THK5351 has high binding affinity and selectivity for tau aggregates [38], although some studies have revealed displacement of [^18^F]-THK5351 binding by selegiline, indicative of monoamine oxidase-B (MAO-B) binding [40]. Increased MAO-B expression is observed in reactive astrogliosis [41] and can therefore also have a neurobiological meaning. The purpose of amyloid PET was to ascertain fibrillary amyloid plaque load. A negative amyloid PET scan in a patient virtually excludes Alzheimer’s disease as the underlying cause [39]. As a secondary objective, performance on domains apart from single-word comprehension, grammatical processing, and motor speech was assessed and compared between other PPA variants.

## Methods

### Subjects

#### Patients

A consecutive series of 21 patients who fulfilled the international consensus criteria for PPA [1] enrolled. Eighteen patients were recruited through the memory clinic University Hospitals Leuven, case 3 was referred by the Free University Amsterdam, and cases 17 and 20 by the University Hospitals Ghent (Table 1). One case (case 15) had to be excluded due to a subarachnoidal cyst anterior to the left temporal lobe. Classification of patients relied on the clinical evaluation by an experienced neurologist in combination with the results from the clinical MRI and 2-[^18^F]-fluoro-2-deoxy-d-glucose PET scan. None of the cases could be considered as primary progressive apraxia of speech [11, 12, 42] since agrammatism was present in all patients who concomitantly exhibited speech apraxia (Table 1). The presence of clinical signs and symptoms on neurological-clinical examination was documented for hypomimetic facies, dysarthria, limb dystonia, extrapyramidal signs, alien limb, nuchal rigidity, diminished postural reflexes, falls, tremor, myoclonus, pyramidal signs, dysphagia, pseudobulbar affect, ideomotor apraxia, and apraxia of eyelid closure (Table 2). A subset of PPA patients (*n* = 13) underwent a cerebrospinal fluid (CSF) Alzheimer’s disease biomarker measurement (Innotest enzyme-linked immunosorbent assay (ELISA) for amyloid-β_1–42_ (Aβ_1–42_; cut-off = 853 pg/ml [43]), total-tau (t-tau; Aβ_1–42_/t-tau cut-off = 2.258), and phospho_181_-tau (p_181_-tau); Fujirebio Europe, Ghent, Belgium), which was performed at the Laboratory of Medical analysis (Medicine Department of UZ Leuven, Leuven, Belgium) as part of the clinical work-up (Table 1). Two cases (case 9 and 14) received [^11^C]-PIB PET as part of the prior clinical work-up [44] (Table 1). In PPA patients who had not previously received an amyloid biomarker measurement, [^11^C]-PIB PET was acquired for the current study.

**Table 1:** Demographics, neurolinguistic, and neuropsychological assessment

	Case																			
	2	4	13	16	17	18	21	3	12	14	19	20	1	5	6	8	10	7	9	11
PPA variant	MV	MV	MV	MV	MV	MV	MV	NFV	NFV	NFV	NFV	NFV	SV	SV	SV	SV	SV	LV	LV	LV
Age (years)	80	62	76	70	65	49	76	57	68	66	63	70	73	71	63	52	55	77	63	74
Gender	M	F	M	M	M	F	F	F	F	F	M	M	F	F	F	F	M	M	F	M
Education (years)	17	12	12	12	15	12	12	16	10	15	10	10	14	8	13	13	14	10	12	18
Handedness	R	L	R	R	R	R	R	R	R	R	R	R	R	R	R	R	R	R	R	R
Symptom duration (months)	33	16	37	39	40	53	24	43	60	74	29	45	19	44	11	131	6	59	95	48
[^11^C]-PIB SUVR	–	1.26	2.1	1.35	1.57	–	–	–	–	1.16	1.37	–	–	–	1.12	1.14	1.2	–	1.81	–
CSF Aβ_1–42_ (pg/ml)	816	–	477	–	759	1077	1144	1057	832	–	–	887	1558	733	–	–	–	564	664	321
CSF t-tau (pg/ml)	195	–	442	–	744	231	265	247	320	–	–	270	428	262	–	–	–	407	–	858
CSF Aβ_1–42_/t-tau	4.18	–	1.08	–	1.02	4.66	4.32	4.28	2.60	–	–	3.29	3.64	2.80	–	–	–	1.39	**–**	0.37
CSF p_181_-tau (pg/ml)	42	–	59.6	–	87.1	31	39.9	34	43	–	–	48	52	36.3	–	–	–	65.7	–	95
CDR	1	0.5	0.5	0.5	1	–	0.50	0.5	0.5	0	0.5	0.5	1	0.5	0.5	0.5	0.5	1	0	0.5
MMSE (/30)^c^	28	28	**24**	**23**	**5**	–	**18**	**24**	**18**	30	26	26	**26**	**25**	29	**23**	30	**26**	29	**24**
CPM (/36)^a^	**26**	31	29	29	**24**	**–**	**17**	**–**	29	**26**	**25**	26	28	27	34	36	34	32	32	**12**
BNT (/60)^b^	52	**46**	**43**	**41**	**4**	**17**	48	**7**	**30**	**47**	53	53	**11**	**14**	**17**	**9**	**33**	46	57	**24**
AVF (1 min)^b^	**8**	**12**	**7**	**9**	**2**	**–**	**5**	**7**	**2**	**13**	16	**4**	**7**	**11**	**13**	**6**	**16**	**7**	23	**8**
AAT sum single-word comprehension (/60)^c^	**41**	**49**	**41**	**42**	**37**	**27**	**47**	56	55	51	53	51	**31**	**32**	**39**	**35**	58	53	54	**50**
PALPA auditory word- picture matching (/40)^c^	40	39	39	39	**38**	**–**	**36**	**37**	39	**38**	40	40	**26**	**27**	**26**	**21**	39	**38**	39	**38**
PALPA verbal assoc.-sem. HI (/15)^c^	**11**	15	12	14	**11**	**–**	14	15	**10**	14	13	**10**	**9**	15	12	**5**	14	15	15	15
PALPA verbal assoc.-sem. LI (/15)^c^	12	**11**	14	**11**	**7**	**–**	**5**	14	**7**	12	**11**	**9**	**3**	**7**	**10**	**6**	14	13	14	12
PPT (/52)^c^	**46**	**47**	**47**	**47**	**47**	**–**	**45**	**47**	**48**	**48**	49	**48**	**31**	**31**	**38**	**34**	**47**	49	52	**45**
BORB easy B (/32)^c^	28	**25**	29	28	29	**–**	27	31	30	28	30	28	**22**	**19**	**22**	**18**	**25**	28	30	26
BORB hard A (/32)^c^	25	**19**	**22**	24	**20**	**–**	**20**	31	**22**	26	**21**	30	**17**	**19**	**22**	**17**	26	23	26	**21**
WEZT verb comprehension (/60)^b^	56	**45**	**55**	**52**	**42**	**–**	**49**	60	**40**	**42**	58	**55**	**43**	**32**	**51**	**48**	58	57	57	**53**
WEZT auditory sentence comprehension (/40)^c^	**36**	**36**	**33**	**31**	**12**	**–**	**23**	**29**	**26**	**35**	**33**	**32**	**37**	**33**	38	38	39	**27**	40	**37**
WEZT active sentence anagram (/10)^c^	10	10	**9**	10	**5**	**–**	10	10	**9**	10	10	10	10	10	10	10	10	10	10	10
WEZT passive sentence anagram (/10)^c^	10	**9**	**9**	**5**	**5**	**–**	**5**	10	**6**	10	10	**3**	10	**9**	10	**9**	10	10	10	10
AAT phoneme repetition (/30)^b^	**24**	**28**	**22**	**20**	**–**	**14**	29	**27**	**27**	30	**27**	**26**	30	29	30	30	30	30	30	29
AAT monosyllabic word repetition (/30)^b^	**28**	30	**19**	30	**–**	**19**	30	29	30	30	**24**	**28**	29	**28**	**27**	30	30	30	30	29
AAT cognate word repetition (/30)^b^	29	30	**16**	29	**–**	**5**	30	**28**	30	30	**29**	**27**	30	30	**28**	30	30	30	**29**	29
AAT concatenated word repetition (/30)^b^	**27**	29	**23**	29	**–**	**1**	30	**15**	29	30	**26**	**25**	30	29	**28**	**26**	30	**15**	29	30
AAT sentence repetition (/30)^c^	**27**	28	28	28	**–**	**0**	**27**	**17**	**24**	28	**25**	**23**	29	29	30	**27**	30	**13**	**26**	**24**
PALPA single-word repetition (/80)^a^	77	80	**55**	77	**–**	**–**	80	76	79	80	**49**	76	79	77	77	80	79	79	80	80
PALPA pseudoword repetition (/80)^a^	**21**	72	**11**	57	**–**	**–**	**53**	63	72	78	**20**	**59**	77	69	67	79	77	66	74	**56**
DIAS diadochokinesis^c^	103	**50**	**42**	115	75	**–**	**32**	**24**	**51**	77	79	**18**	**50**	70	147	125	80	77	114	117
DIAS consonant and vowel repetition (/30)^c^	**28**	**24**	**12**	**27**	**11**	**–**	**28**	**28**	**20**	30	30	**25**	**27**	**24**	30	30	30	30	30	**29**

Data in bold are abnormal based on a Crawford and Garthwaite [64] regression method, correcting for education^a^ or correcting for age^b^ or depending on the outcome of a Crawford and Howell [52] modified *t* test^c^

*AAT* Aachen Aphasia Test, *assoc.-sem* associative semantic, *Aβ*_*1–42*_ amyloid-β_1–42_, *AVF* Animal Verbal Fluency, *BNT* Boston Naming Test, *BORB* Birmingham Object Recognition Battery, *CDR* Clinical Dementia Rating, *[*^*11*^*C]-PIB* [^11^C]-Pittsburgh Compound B, *CPM* Colored Progressive Matrices, *CSF* cerebrospinal fluid, *DIAS* Diagnostisch Instrument voor Apraxie van de Spraak, *F* female, *HI* high imageability, *L* left-handed, *LI* low imageability, *LV* logopenic variant, *M* male, *MMSE* Mini-Mental State Examination, *MV* mixed variant, *NFV* nonfluent variant, *PALPA* Psycholinguistic Assessment of Language Processing in Aphasia, *PPA* primary progressive aphasia, *PPT* Pyramids and Palm trees Test, *p181-tau* phospho181-tau, *R* right-handed, *SUVR* standardized uptake value ratio in a composite cortical volume of interest, *SV* semantic variant, *t-tau* total-tau, *WEZT* Werkwoorden En Zinnen test

– no data collected.

**Table 2:** Clinical signs and symptoms in primary progressive aphasia (PPA)

Case	2	4	13	16	17	18	21	3	12	14	19	20	1	5	6	8	10	7	9	11
PPA variant	MV	MV	MV	MV	MV	MV	MV	NFV	NFV	NFV	NFV	NFV	SV	SV	SV	SV	SV	LV	LV	LV
Hypomimetic facies	–	+	–	–	–	–	–	–	+	–	–	–	–	–	–	–	–	–	–	–
Dysarthria	–	+	–	–	–	–	+	–	+	–	–	–	–	–	–	–	–	–	–	–
Right-sided limb dystonia	–	+	–	–	–	–	–	–	–	–	–	–	–	–	–	–	–	–	–	–
Right-sided extrapyramidal signs	–	+	+	–	–	–	–	–	+	+	+	+	–	–	–	–	–	–	–	–
Alien limb	–	–	–	–	–	–	–	–	+	–	–	–	–	–	–	–	–	–	–	–
Nuchal rigidity	–	+	+	–	–	–	+	–	–	–	–	–	–	–	–	–	–	–	–	–
Reduced postural reflexes	–	+	–	–	–	–	–	–	–	–	–	–	–	–	–	–	–	–	–	–
Falls	+	+	–	–	–	–	–	–	+	–	–	–	–	–	–	–	–	–	–	–
Tremor	–	–	+	–	–	–	–	–	+	–	–	–	–	–	–	–	–	–	–	–
Myoclonus	–	–	–	–	–	–	–	–	–	–	–	–	–	–	–	–	–	–	–	–
Vertical gaze slowing or palsy	–	+	+	+	–	–	+	–	+	+	–	+	–	–	–	–	–	–	–	–
Decrease in vertical optokinetic nystagmus	+	+	+	+	–	–	+	–	+	+	–	+	–	–	–	–	–	–	–	–
Pyramidal signs	–	–	–	–	–	–	–	+	+	–	–	–	–	–	–	–	–	–	–	–
Dysphagia	+	+	–	–	–	–	–	–	–	–	–	–	–	–	–	–	–	–	–	–
Pseudobulbar affect	+	–	–	–	–	–	–	–	–	–	–	–	–	–	–	–	–	–	–	–
Ideomotor apraxia	–	–	–	–	–	–	–	–	–	–	–	–	–	–	–	–	–	–	–	–
Apraxia of eyelid closure	–	–	–	–	–	–	–	–	–	–	–	–	–	–	–	–	–	–	–	–

*LV* logopenic variant, *MV* mixed variant, *NFV* nonfluent variant, *SV* semantic variant

### Control subjects

For normative reasons, data of four groups of cognitively intact older healthy controls were used (Additional file 1: Table S1). Inclusion criteria for controls were Mini-Mental State Examination (MMSE) [45] score ≥ 27, a Clinical Dementia Rating (DCR) scale [46] global score of zero, and no history of neurological or psychiatric disease or brain lesions on structural MRI [44, 47–49]. The neuropsychological data of the first group of 64 healthy controls were used to calculate whether neuropsychological and language performance of an individual PPA patient was within normal limits (Additional file 1: Table S1). Of these 64 control subjects, 20 also underwent [^18^F]-THK5351 PET in the context of the current study, 22 underwent high resolution T_1_-weighted structural MRI on the same scanner as patients, and 14 of these 20 subjects (six had to be excluded due to claustrophobia or movement) also underwent [^11^C]-PIB PET as part of the current study protocol. Of these 64 control subjects, 16 additional subjects had also received an amyloid PET scan for other studies which was negative on visual assessment [47].

For the purpose of comparing gray matter volume in PPA patients with healthy controls, 19 additional high-resolution T_1_-weighted structural MRI scans of a second healthy control group were selected, resulting in a total control group of 41 MRI scans for gray matter volumetric analyses (Additional file 1: Table S1). These 41 cognitively intact older controls were amyloid-negative based on visual assessment.

For the purpose of comparing [^11^C]-PIB binding, [^11^C]-PIB scans of a group of 19 additional amyloid-negative healthy controls were used [48], leaving a total group of 33 [^11^C]-PIB scans (Additional file 1: Table S1). Amyloid-negativity of these controls was assured visually and using a semiquantitative cut-off as described previously [48].

### Neuropsychological and neurolinguistic protocol

General cognitive functioning was assessed by CDR and MMSE [45]. Colored Progressive Matrices (CPM) were used to assess nonverbal fluid intelligence. Confrontation naming was assessed by means of the Boston Naming Test (BNT) [50], a 60-item standardized test in which items are administered in order of decreasing frequency of occurrence in the language. Category verbal fluency was assessed by the 1-min Animal Verbal Fluency (AVF) test.

The main aim was to study single-word comprehension problems in patients with speech apraxia and/or agrammatism. Single-word comprehension was assessed using the Dutch version of the Aachen Aphasia Test (AAT) [51]. Performance on auditory and written single-word comprehension was considered as one ‘sum’ score (Tables 1 and 3). In both the auditory and written single-word comprehension subtests of the AAT, 10 words are presented per modality. One target picture and three distracter pictures are presented simultaneously, and subjects have to indicate the picture that corresponds to the word. One distracter picture is semantically related to the target picture. Interpretation of an individual patient’s performance on the sum of the AAT auditory and written single-word comprehension test was statistically compared with the healthy control group (Additional file 1: Table S1) based on a modified *t* test [52] (see Statistical analyses).

**Table 3:** Statistical group comparisons of neurolinguistic and neuropsychological data

	Group							MV comparisons^c^				Other comparisons^c^			
Variables	MV	NFV	SV	LV	HC	N HC	*P* ^a,b^	MV-HC	MV-NFV	MV-SV	MV-LV	NFV-HC	NFV-SV	SV-HC	LV-HC
Age (years)	70 (49–80)	66 (57–70)	63 (52–73)	74 (63–77)	67.5 (53–89)	64	0.50^a^	–	–	–	–	–	–	–	–
Gender (female/male)	3/4	3/2	4/1	1/2	34/30	64	0.68^b^	–	–	–	–	–	–	–	–
Education (years)	12 (12–17)	10 (10–16)	13 (8–14)	12 (10–18)	13 (8–22)	64	0.76 ^a^	–	–	–	–	–	–	–	–
Symptom duration (months)	37 (16–53)	45 (29–74)	19 (6–131)	59 (48–95)	–	–	0.11^a^	–	–	–	–	–	–	–	–
CDR	0.5 (0.5–1)	0.5 (0–0.5)	0.5 (0.5–1)	0.5 (0–1)	0 (0)	64	**< 0.001** ^**a**^	**< 0.001**	0.064	0.43	0.45	**< 0.001**	0.18	**< 0.001**	**< 0.001**
MMSE (/30)	23.5 (5–28)	26 (18–30)	26 (23–30)	26 (24–29)	29 (27–30)	64	**0.002** ^**a**^	**0.001**	0.46	0.17	0.24	**0.015**	0.67	0.07	**0.04**
CPM (/36)	27.5 (17–31)	26 (25–29)	34 (27–36)	32 (12–32)	33 (24–36)	62	**0.001** ^**a**^	**0.001**	0.83	0.099	0.44	**0.003**	**0.048**	0.70	0.15
BNT (/60)	43(4–52)	47 (7–53)	14 (9–33)	46 (24–57)	56 (44–60)	64	**< 0.001** ^**a**^	**< 0.001**	0.46	0.074	0.49	**0.005**	0.17	**< 0.001**	0.16
AVF (1 min)	7.5 (2–12)	7 (2–16)	11 (6–16)	8 (7–23)	22.0 (14–30)	35	**< 0.001** ^**a**^	**< 0.001**	0.85	0.23	0.44	**0.001**	0.53	**0.001**	0.087
AAT sum single-word comprehension (/60)	41 (27–49)	53 (51–56)	35 (31–58)	53 (50–54)	58 (48–60)	62	**0.001** ^**a**^	**< 0.001**	**0.004**	0.37	**0.016**	**0.011**	0.12	**0.003**	**0.023**
PALPA auditory word-picture matching (/40)	39 (36–40)	39 (37–40)	26 (21–39)	38 (38–39)	40 (38–40)	49	**< 0.001** ^**a**^	**0.001**	0.71	**0.031**	0.49	**0.030**	**0.035**	**< 0.001**	**0.001**
PALPA verbal assoc.-sem. HI (/15)	13 (11–15)	13 (10–15)	12 (5–15)	15 (15–15)	15 (10–15)	51	**0.004** ^**a**^	**0.02**	0.64	0.58	**0.041**	**0.025**	0.60	**0.018**	0.17
PALPA verbal assoc.-sem. LI (/15)	11 (5–14)	11 (7–14)	7 (3–14)	13 (12–14)	13 (11–15)	51	**< 0.001** ^**a**^	**0.003**	0.78	0.36	0.12	**0.009**	0.25	**0.006**	0.46
PPT (/52)	47 (45–47)	48 (47–49)	34 (31–47)	49 (45–52)	51 (45–52)	60	**< 0.001** ^**a**^	**< 0.001**	**0.012**	**0.035**	0.34	**< 0.001**	**0.011**	**< 0.001**	0.23
BORB easy B (/32)	28 (25–29)	30 (28–31)	22 (18–25)	28 (26–30)	30 (25–32)	62	**< 0.001** ^**a**^	**0.017**	0.091	**0.008**	0.79	0.68	**0.008**	**< 0.001**	0.17
BORB hard A (/32)	21 (19–25)	26 (21–31)	19 (17–26)	23 (21–26)	27 (20–31)	62	**< 0.001** ^**a**^	**< 0.001**	0.081	0.36	0.30	0.79	0.073	**0.001**	**0.034**
WEZT verb comprehension (/60)	50.5 (42–56)	55 (40–60)	48 (32–58)	57 (53–57)	59 (56–60)	21	**< 0.001** ^**a**^	**< 0.001**	0.71	0.58	0.070	**0.037**	0.53	**0.001**	**0.013**
WEZT auditory sentence comprehension (/40)	32 (12–36)	32 (26–35)	38 (33–39)	37 (27–40)	40 (36–40)	23	**< 0.001** ^**a**^	**< 0.001**	0.93	**0.022**	0.20	**< 0.001**	**0.021**	**0.001**	0.097
WEZT active sentence anagram (/10)	10 (5–10)	10 (9–10)	10 (10–10)	10 (10–10)	10 (10–10)	23	**0.046** ^**a**^	**0.005**	0.56	0.18	0.29	**0.032**	0.32	1	1
WEZT passive sentence anagram (/10)	7 (5–10)	10 (3–10)	10 (9–10)	10 (10–10)	10 (10–10)	23	**< 0.001** ^**a**^	**< 0.001**	0.40	0.066	**0.038**	**0.002**	0.64	**0.002**	1
AAT phoneme repetition (/30)	23 (14–29)	27 (26–30)	30 (29–30)	30 (29–30)	30 (28–30)	49	**< 0.001** ^**a**^	**< 0.001**	0.20	**0.007**	**0.027**	**< 0.001**	**0.032**	0.93	0.55
AAT monosyllabic word repetition (/30)	29 (19–30)	29 (24–30)	29 (27–30)	30 (29–30)	30 (27–30)	49	0.076^a^	**–**	–	–	–	**–**	–	–	–
AAT cognate word repetition (/30)	29 (5–30)	29 (27–30)	30 (28–30)	30 (29–30)	30 (27–30)	49	**0.002** ^**a**^	**< 0.001**	0.71	0.16	0.27	**0.002**	0.24	0.46	0.24
AAT concatenated word repetition (/30)	28 (1–30)	26 (15–30)	29 (26–30)	29 (15–30)	30 (28–30)	49	**0.001** ^**a**^	**0.001**	0.93	0.35	0.69	**0.002**	0.24	0.058	0.088
AAT sentence repetition (/30)	27.5 (0–28)	24 (17–28)	29 (27–30)	24 (13–26)	30 (26–30)	49	**< 0.001** ^**a**^	**< 0.001**	0.22	**0.04**	0.11	**< 0.001**	**0.016**	0.33	**0.001**
PALPA single-word repetition (/80)	77 (55–80)	76 (49–80)	79 (77–80)	80 (79–80)	80 (73–80)	50	0.10^a^	**–**	–	–	–	**–**	–	–	–
PALPA pseudoword repetition (/80)	53 (11–72)	63 (20–78)	77 (67–79)	66 (56–74)	77 (45–80)	50	**0.001** ^**a**^	**0.001**	0.21	**0.028**	0.18	**0.022**	0.17	0.53	**0.035**
DIAS diadochokinesis	62.5 (32–115)	51 (18–79)	80 (50–147)	114 (77–117)	126 (56–192)	23	**0.001** ^**a**^	**0.003**	0.47	0.23	0.12	**0.001**	0.12	0.093	0.10
DIAS consonant and vowel repetition (/30)	25.5 (11–28)	28 (20–30)	30 (24–30)	30 (29–30)	30 (29–30)	23	**< 0.001** ^**a**^	**< 0.001**	0.20	0.10	**0.019**	**0.008**	0.58	0.096	0.37

Data are shown as median and range (minimum–maximum)

*AAT* Aachen Aphasia Test, *assoc.-sem* associative semantic, *AVF* Animal Verbal Fluency, *BNT* Boston Naming Test, *BORB* Birmingham Object Recognition Battery, *CDR* Clinical Dementia Rating, *CPM* Colored Progressive Matrices, *DIAS* Diagnostisch Instrument voor Apraxie van de Spraak, *HI* high imageability, *LI* low imageability, *LV* logopenic variant, *MMSE* Mini-Mental State Examination, *MV* mixed variant, *NFV* nonfluent variant, *PALPA* Psycholinguistic Assessment of Language Processing in Aphasia, *PPT* Pyramids and Palm trees Test, *SV* semantic variant, *WEZT* Werkwoorden En Zinnen test

– Data not available

^a^Kruskal-Wallis statistical analyses were performed to assess between-group differences (HC, MV, NFV, SV and LV) for continuous variables and ^b^chi-square tests for categorical variables. Pair-wise post-hoc comparisons were performed with Mann-Whitney *U* tests^c^. *P* values are not corrected for multiple comparisons. *P* values in bold are significantly different between groups

The Dutch version of the Psycholinguistic Assessment of Language Processing in Aphasia (PALPA) [53] auditory word-picture matching task (PALPA subtest 45) was additionally used to assess single-word comprehension deficits. In this task (40 trials), a concrete noun is presented auditorily together with a target picture and four distractors. Two distractors are semantically related to the target, a third is perceptually similar, and the fourth picture is unrelated. Subjects were asked to point to the target picture.

In the PALPA associative-semantic task (PALPA subtest 49), a noun is presented visually together with four choice noun stimuli (a target noun, a noun that is semantically related to the target, and two unrelated nouns). Subjects have to underline or circle the noun that matches the sample stimulus most closely in meaning, for a total of a 15-word series with high imageability and 15 with low imageability. Associative-semantic memory was also assessed by the picture-version of the Pyramids and Palm trees Test (PPT) (52 trials) [54] which relies on the capability to make semantic associations between two pictures. The Birmingham Object Recognition Battery (BORB) (easy (B) and hard (A), 32 trials each) [55] was used as a measure of object identification. In this task, subjects have to indicate whether an animal or tool depicted in a line drawing is real or unreal.

Verb comprehension and grammaticality were assessed by the Werkwoorden En Zinnen Test (WEZT) [56]. Grammaticality was quantified using both the WEZT auditory sentence comprehension test (40 trials) as well as the WEZT sentence anagram test (20 trials). During administration of the WEZT sentence anagram test, the patient is asked to manually put together single words which are each printed on separate cards into a syntactic structure that describes the action depicted in the target picture. Sentences can occur in the active or passive tense, of which half represent reversible actions and half represent irreversible actions.

In the AAT repetition task, the examiner pronounces 10 phonemes, 10 monosyllabic words, 10 cognate (foreign) words, 10 composed (concatenated) words, and 10 sentences of increasing length which the subject has to repeat. In the PALPA word repetition task (PALPA subtest 9), the examiner who is sitting in front of the subject pronounces 80 nouns and 80 pseudowords which the subject has to repeat.

The Diagnostisch Instrument voor Apraxie van de Spraak (DIAS) [57] was used to assess consonant and vowel repetition (15 trials each), of which the sum is considered the ‘DIAS severity score’. Diadochokinesis, which is the ability to make antagonistic movements using different parts of the mouth, tongue, and soft palate in quick succession, was also assessed using DIAS. During this DIAS diadochokinesis task, the examiner first reads three successive sounds or tokens aloud, e.g., the alternating task ‘pa ta ka’ or the sequential task ‘pa pa pa’, and asks the patient to repeat these once. If the patient was able to repeat this sequence correctly, he/she was asked to repeat it as many times as possible during a period of s-8. In total, the patient has to repeat six alternating and six sequential sounds, for which the total number of repetitions was scored (Tables 1 and 3).

### [^18^F]-THK5351 PET acquisition and analysis

[^18^F]-THK5351 PET scans were acquired on a 16-slice Siemens Biograph PET/computed tomography (CT) scanner (Siemens Medical Solutions, Erlangen, Germany) in 20 patients and in 20 healthy control subjects (Additional file 1: Table S1). After bolus injection of [^18^F]-THK5351 (mean dose = 185.2 MBq, range 178.7–191.0 MBq) in an antecubital vein, five healthy control subjects were scanned dynamically with arterial sampling between 0 and 100 min postinjection to assess the optimal PET imaging window. The remaining healthy controls and all PPA patients were scanned between 50 and 80 min postinjection with [^18^F]-THK5351 (controls: mean dose = 184.1 MBq, range 165.8–196.0 MBq; patients: mean dose = 181.8 MBq, range 164.9–192.3 MBq). A low-dose CT scan was acquired for attenuation correction prior to PET scan acquisition. PET emission images were acquired in 3D mode and subsequently reconstructed using ordered subsets expectation maximization (4 iterations × 16 subsets). Individual [^18^F]-THK5351 PET emission frames were realigned to correct for head-motion, summed, and rigidly coregistered to the subject’s T_1_-weighted MRI scan using Statistical Parametric Mapping software (SPM12, Wellcome Trust Centre for Neuroimaging, London, UK) implemented in Matlab R2014b (Mathworks, Natick, USA). Summed PET images as well as the T_1_-weighted MRI segmentations were warped to Montreal Neurological Institute (MNI) template space. The normalized [^18^F]-THK5351 PET scans were subsequently corrected for partial volume effects using the modified Müller-Gärtner method [58]. Partial volume corrected (PVC) standardized uptake value ratio (SUVR) images with the subject-specific cerebellar gray matter as reference region were created. For voxel-based statistical analyses, PVC [^18^F]-THK5351 SUVR images were smoothed with an isotropic 8-mm full-width half-maximum (FWHM) Gaussian kernel. The [^18^F]-THK5351 PET data were obtained within 2–182 days from the [^11^C]-PIB PET scan (mean 82 days in controls, mean 19 days in PPA patients).

### Volumetric MRI acquisition and analysis

All subjects received MRI scanning on the same day as the neuropsychological testing. The high-resolution T_1_-weighted structural MRI scan was acquired on a 3-Tesla Philips Achieva dstream equipped with a 32-channel head volume coil (Philips, Best, The Netherlands), using a 3D turbo field echo sequence (coronal inversion recovery prepared 3D gradient-echo images, inversion time (TI) 900 ms, shot interval = 3000 ms, echo time (TE) = 4.6 ms, repetition time (TR) = 9.6 ms, flip angle 8 degrees, field of view (FoV) = 250 × 250 mm, 182 slices, slice thickness = 1.2 mm, voxel size = 0.98 × 1.2 × 0.98 mm^3^).

All T_1_-weighted images underwent preprocessing with voxel-based morphometry (VBM8) [59] as previously described [60, 61]. This included corrections for gradient nonlinearity and intensity inhomogeneity in the MRI. The resulting modulated gray matter volumes were adjusted for overall brain size (total intracranial volume) by using the ‘nonlinear-only’ component in the spatial normalization process for modulation of gray matter voxel intensities [62]. For voxel-based statistical analyses, modulated gray matter maps were smoothed with an 8-mm FWHM Gaussian 3D kernel.

### Amyloid biomarker measurement and analysis

[^11^C]-PIB PET scans were acquired on a GE Signa 3-T PET/MRI scanner (GE Healthcare, Chicago, USA) operating in 3D mode to estimate amyloid burden in eight patients and 14 healthy control subjects. [^11^C]-PIB was injected intravenously as a bolus in an antecubital vein (controls: mean dose = 267.8 MBq, range 197.5–364.9 MBq; patients: mean dose = 270.5 MBq, range 230.6–316.4 MBq). Dynamic [^11^C]-PIB images were acquired during a 70-min period and reconstructed with atlas-based attenuation correction using the manufacturer’s software. Two patients received a 30-min [^11^C]-PIB PET scan between 40 and 70-min postinjection on a Siemens Biograph PET/CT scanner (Siemens Medical Solutions, Erlangen, Germany) in a clinical context (See Subjects section). For the purpose of comparing [^11^C]-PIB binding in these two patients, [^11^C]-PIB scans acquired on the same Siemens Biograph PET/CT scanner in 19 older amyloid-negative cognitively intact control subjects were used (Additional file 1: Table S1) [48]. Processing of [^11^C]-PIB PET images was performed in SPM12 using the same MRI-based method as previously described [48]. [^11^C]-PIB PET images were corrected for partial volume effects using a modified Müller-Gärtner method [58]. The mean [^11^C]-PIB PET SUVR value was calculated in a neocortical composite region [63] and considered positive if this value was significantly elevated compared with healthy controls based on a modified *t* test [52]. For voxel-based statistical analyses, PVC [^11^C]-PIB SUVR images were smoothed with an isotropic 6-mm FWHM Gaussian kernel.

### Statistical analyses

#### Neuropsychological and neurolinguistic data analyses

Standard statistical analyses were performed in Statistics Software Package for the Social Sciences (version 24, IBM Statistics, Armonk, USA). The significance was set at *P* < 0.05 for all standard statistical analyses. Demographic data were statistically compared using Kruskal-Wallis for continuous variables and using Pearson chi-squared tests for categorical variables.

A first objective was to assess the prevalence of single-word comprehension deficits in individual PPA patients with speech apraxia and/or agrammatism. The neuropsychological test scores of individuals were statistically contrasted with scores derived from 64 healthy controls (Additional file 1: Table S1) following the procedure developed by Crawford and Garthwaite [64]. As a first step, a hierarchical multiple linear regression analysis was performed in the healthy control group for each task, with the neuropsychological test scores as outcome variable and age and education as predictor variables. Variables that had a significant effect (i.e. α < 0.05) on any of the test scores in the healthy controls were included as predictor variables in the regression equation for that test.

There was a statistically significant positive effect of education on performance on the CPM task (*R*^*2*^ adjusted = 0.069, *P* = 0.022) and on PALPA repetition of words (*R*^*2*^ adjusted = 0.11, *P* = 0.009) and pseudowords (*R*^*2*^ adjusted = 0.11, *P* = 0.013). Age had a significant negative effect on performance on the BNT (*R*^*2*^ adjusted = 0.047, *P* = 0.046), AVF (*R*^*2*^ adjusted = 0.19, *P* = 0.005), WEZT verb comprehension (*R*^*2*^ adjusted = 0.15, *P* = 0.045), AAT repetition of phonemes (*R*^*2*^ adjusted = 0.068, *P* = 0.039), of single words (*R*^*2*^ adjusted = 0.065, *P* = 0.043), cognates (*R*^*2*^ adjusted = 0.15, *P* = 0.003), and concatenated words (*R*^*2*^ adjusted = 0.11, *P* = 0.011). Consequently, education or age, respectively, were entered in the regression equation as predictor variables and the test score of the individual patient as a dependent variable in order to obtain a predicted score for that individual patient. The discrepancy between the predicted score and the observed score was expressed as a *Z* score. Individual performance on the other tasks was statistically compared with the healthy control group based on a modified *t* test [52]. The corresponding *P* values were converted to a *Z* score. For all tests, *Z* scores were considered abnormal at 1.96 standard deviations [65] (Table 1).

The nature of the single-word comprehension deficit in MV PPA was subsequently analyzed in detail by assessing the influence of word frequency and the effect of the number of phonemes contained in a word (phonemic length). Word frequency was retrieved using the SUBTLEX-NL database for the dominant meaning of words [66]. In 11 out of 20 trials of the AAT auditory and written single-word comprehension test, the nondominant meaning of a word with multiple meanings (i.e., a homonym) is targeted. For instance, in case the word ‘star’ is presented to the subject, the target picture depicts a ‘popstar’ and the distractor picture depicts a ‘sun’, which is semantically related to the dominant meaning but not to the nondominant meaning of ‘star’. To estimate the relative meaning frequencies of each meaning of a homonym, we made use of lexical associations as described in Armstrong et al. [67]. Associations of the words used in the AAT single-word comprehension task were taken from the Small World of Words Project [68]. The estimated frequency of the nondominant meaning of a word was calculated by multiplying the relative frequency with the frequency retrieved from the SUBTLEX-NL database. The frequency of the words assessed in the PALPA word-picture matching task (PALPA subtest 45) were directly taken from the SUBTLEX-NL database, as only the dominant meaning of words was targeted in this test. In total, we were able to retrieve word frequency for 53 out of 60 words from the pooled AAT and PALPA single-word comprehension tasks. For these 53 words, phonemic length was calculated by counting the numbers of phonemes contained in a word. The effects of word frequency and phonemic length on single-word comprehension was assessed by calculating a logistic regression equation for each patient with the patient’s response (correct/not correct) as a dependent variable and word frequency and phonemic length as independent variables [69]. The individual β coefficients derived from this regression equation were compared between MV and SV PPA using two-tailed Mann-Whitney *U* tests with significance set at α < 0.05. The effect of ‘meaning dominance’ on single-word comprehension was calculated by dividing the errors made on trials targeting the nondominant meaning of a word by the total number of errors and this proportion was compared between MV and SV PPA using two-tailed Mann-Whitney *U* tests with significance set at α < 0.05.

As a secondary objective, neuropsychological performance in domains apart from the defining domains of single-word comprehension, grammatical processing, and motor speech was compared to healthy controls, NFV pure, SV, and LV PPA using Kruskal-Wallis, followed by two-tailed Mann-Whitney *U* post-hoc tests with significance set at *P* < 0.05.

### Imaging-based analyses

At the group-level, gray matter volume images, [^18^F]-THK5351, and [^11^C]-PIB PET PVC SUVR images of MV PPA patients were compared with healthy control subjects, NFV pure, SV, and LV PPA patients (between-subjects factor) using separate whole-brain voxel-wise analyses of variance (ANOVA) with two-tailed post-hoc *t* tests in SPM12 running on Matlab R2014b (Mathworks, Natick, USA) with age and gender as nuisance variables. For the ANOVA with [^11^C]-PIB PET SUVR images, scanner type was additionally added as a nuisance variable. The default significance threshold was set at voxel-level uncorrected *P* < 0.001, with a cluster-level family wise error (FWE)-corrected threshold of *P* < 0.05 [70]. At the individual patient level, imaging data were statistically contrasted with the healthy control group for each imaging modality using a voxel-wise modified *t* test [52] at a voxel-level uncorrected *P* < 0.001.

## Results

Groups were matched for age, education, and gender (Table 3), and PPA patients did not differ in symptom duration (Table 3).

### Neuropsychological, neurolinguistic, and clinical-neurological profile of individual MV PPA patients

Significant deficits at the individual patient level are shown in bold in Table 1. Twelve cases fulfilled a priori diagnostic criteria for NFV PPA encompassing agrammatism in language production and/or apraxia of speech presenting as effortful, halting speech with inconsistent speech sound errors and distortions [1] as measured by the repetition tests of AAT and PALPA subtest 9 along with the DIAS and WEZT (Table 1). In these NFV PPA patients, syntactic comprehension measured with the WEZT auditory sentence comprehension test was additionally affected, and object identification measured with the BORB object recognition task was preserved (i.e., fulfilling two out of three ancillary features) [1]. In one NFV case (case 14) agrammatism was the most prominent clinical abnormality without features of apraxia of speech. Seven out of the 12 cases classified a priori as NFV PPA had concomitant single-word comprehension problems as measured with the sum of the AAT auditory and written single-word comprehension test (Table 1). Hence, these patients did not strictly fulfill criteria for NFV pure nor for any of the other PPA variants [1]. This phenotype was in accordance with a diagnosis of MV PPA [19, 20]. These MV PPA cases included three females and four males, with a mean age of onset of 65 years (range 44–77 years).

At the time of testing, mild right-sided extrapyramidal signs were present in two of the MV cases (cases 4 and 13) and in four of the pure NFV cases (cases 12, 14, 19, and 20) (Table 2). Five of the MV cases (cases 2, 4, 13, 16, and 21) and three of the NFV cases (cases 12, 14, and 20) showed mild vertical eye movement abnormalities (Table 2).

### Detailed assessment of single-word comprehension problems

**Fig. 1:**
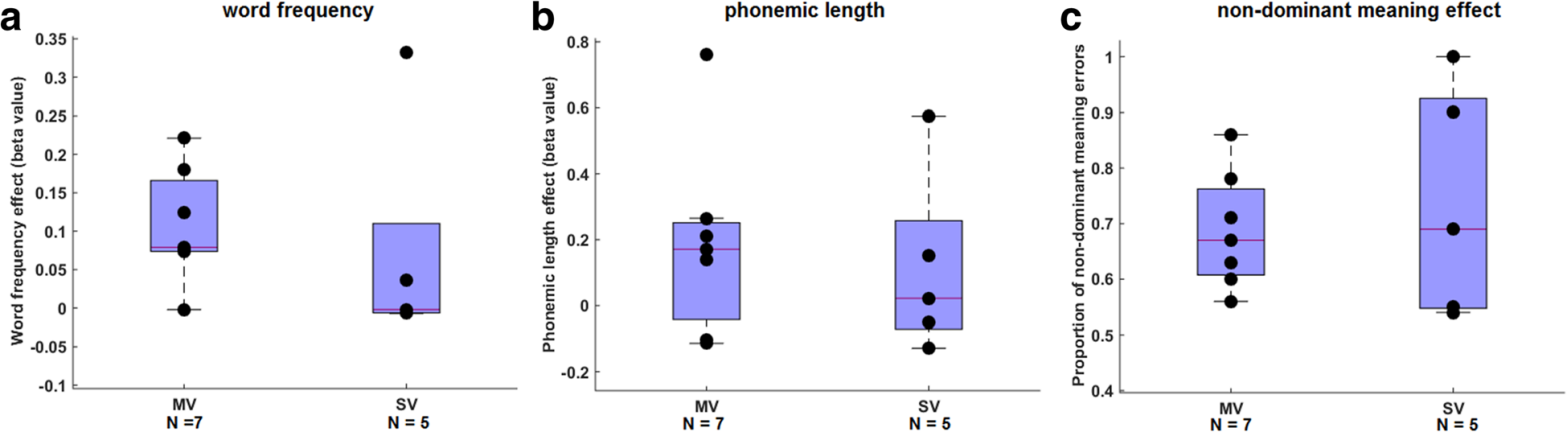
Single-word comprehension error typing in mixed variant (MV) compared with semantic variant (SV) PPA. **a** Word frequency, **b** phonemic length, and **c** proportion of errors on trials targeting the nondominant meaning of a word. Boxes of the boxplots represent 25th, 50th, and 75th percentiles. Individual subject points are shown in black but might overlap

In none of the individual MV or SV cases was a significant effect of word frequency or phonemic length on single-word comprehension present. Word frequency effects on single-word comprehension in MV were not significantly different compared with SV PPA (*U* = 8.5, *P* = 0.14) (Fig. 1a), nor did effects of phonemic length on single-word comprehension differ between MV and SV PPA (*U* = 13, *P* = 0.47) (Fig. 1b). A detailed assessment of the type of single-word comprehension deficits in MV PPA patients revealed that 69% of errors were made on trials assessing the nondominant meaning of a homonym (Fig. 1c). In these trials, patients were not able to retrieve the nondominant meaning of a word but pointed to the distracter picture, which was semantically related to the dominant meaning of that word. The proportion of errors on trials targeting the nondominant meaning of a word did not significantly differ between MV and SV PPA (*U* = 17, *P* = 0.94) (Fig. 1c).

### Group-based deficits of MV PPA for additional neuropsychological domains

**Fig. 2:**
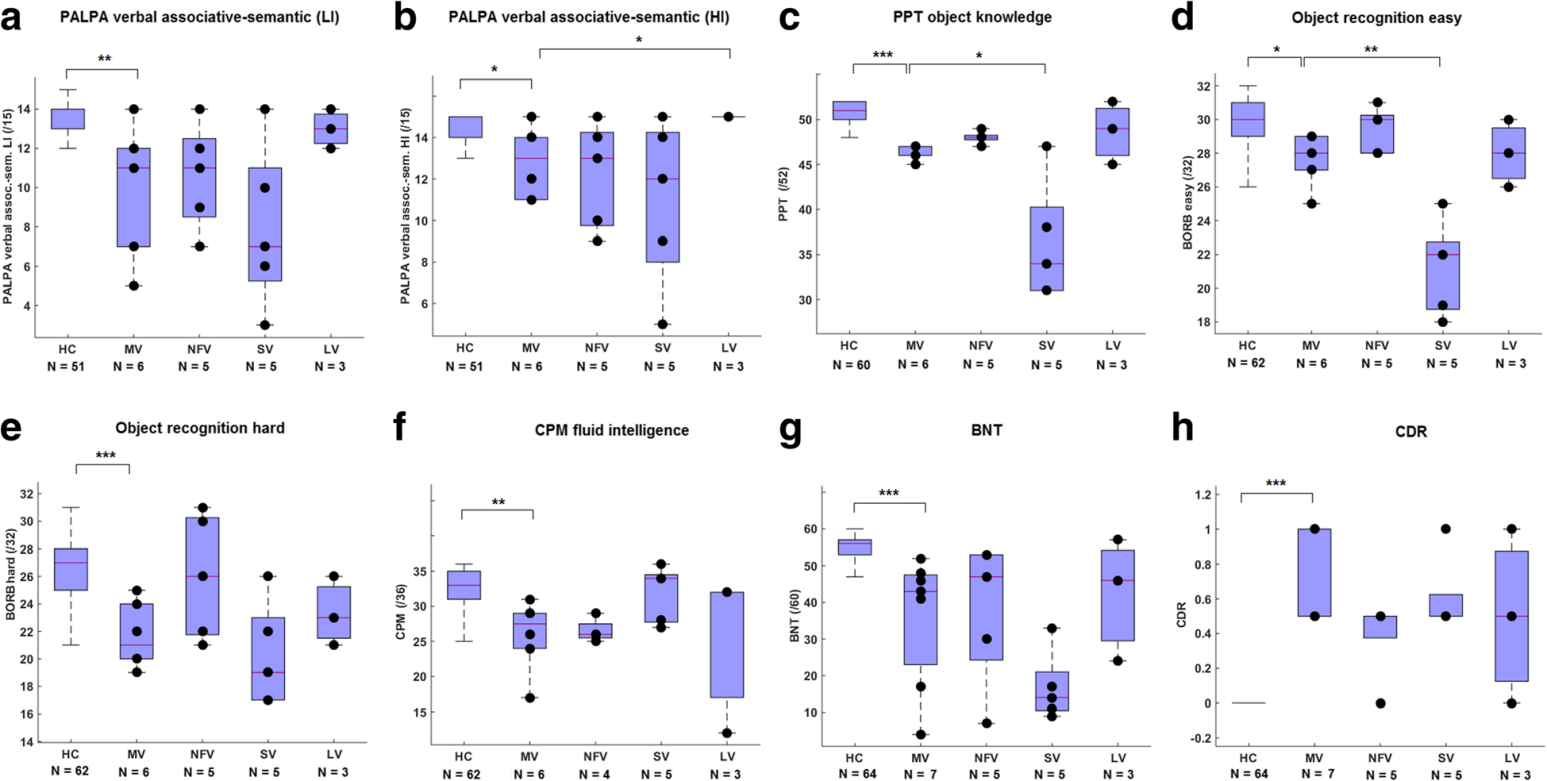
Neuropsychological performance in MV PPA. Statistically significant differences between mixed variant (MV) and the other groups (healthy controls (HC), nonfluent/agrammatic variant (NFV) pure, semantic variant (SV), and logopenic variant (LV)) are indicated. **a,b** Psycholinguistic Assessment of Language Processing in Aphasia (PALPA) associative-semantic test (subtest 49), **c** Pyramids and Palm trees Test (PPT), **d** Birmingham Object Recognition Battery (BORB) easy, **e** BORB hard, **f** Colored Progressive Matrices (CPM)**, g** Boston Naming Test (BNT), and **h** Clinical Dementia Rating (CDR) global score. Boxes of the boxplots represent 25th, 50th, and 75th percentiles. Individual subject points are shown in black but might overlap. *p < 0.05, **p < 0.01, ***p < 0.001. HI high imageability, LI low imageability

Deficits in MV PPA were present in other neuropsychological domains apart from grammatical, motor speech, or single-word comprehension tasks (Fig. 2) (Table 3). The full statistical results for all tasks are shown in Table 3.

Scores on the PALPA verbal associative-semantic task were significantly lower in the MV PPA group compared with healthy controls for words with low (*U* = 40.5, *P* = 0.003) as well as high imageability (*U* = 72, *P* = 0.020) (Fig. 2a, b). These scores were lower in MV compared with LV for the high imageability task (*U* = 1.5, *P* = 0.041) (Fig. 2b). Scores on the picture-version of the PPT were significantly lower in MV PPA compared with healthy controls (*U* = 5.5, *P* < 0.001) and were significantly higher compared with SV PPA (*U* = 4.0, *P* = 0.035) (Fig. 2c). Scores on the BORB object recognition task were significantly lower in MV PPA compared with healthy controls for the easy task (*U* = 77, *P* = 0.017) and for the hard task (*U* = 26, *P* < 0.001) (Fig. 2d, e). However, MV patients scored better than SV on the easy BORB object recognition task (*U* = 0.5, *P* = 0.008) (Fig. 2d), but not on the hard BORB object recognition task (*P* = 0.36) (Fig. 2e). No differences were found between MV and NFV pure and between MV and LV on the BORB object recognition tasks (*P* > 0.081) (Fig. 2d, e) (Table 3).

Compared with healthy controls, MV patients had significantly lower scores on nonverbal fluid intelligence measured by the CPM (*U* = 38.5, *P* = 0.001). No differences were found compared with other variants (*P* > 0.099) (Fig. 2f) (Table 3).

Scores on the BNT were significantly lower in MV PPA compared with healthy controls (*U* = 22.5, *P* < 0.001), but did not differ compared with the other variants (*P* > 0.074) (Fig. 2g) (Table 3).

The global CDR score was significantly higher in MV PPA compared with healthy controls (*U* = 0, *P* < 0.001), with no differences in global CDR score compared with the other PPA variants (*P* > 0.064) (Fig. 2h) (Table 3). Despite the single-word comprehension deficit measured on AAT in MV, performance on the PALPA auditory word-picture matching was less affected in MV compared with SV (Table 3).

### Anatomy of atrophy

**Fig. 3:**
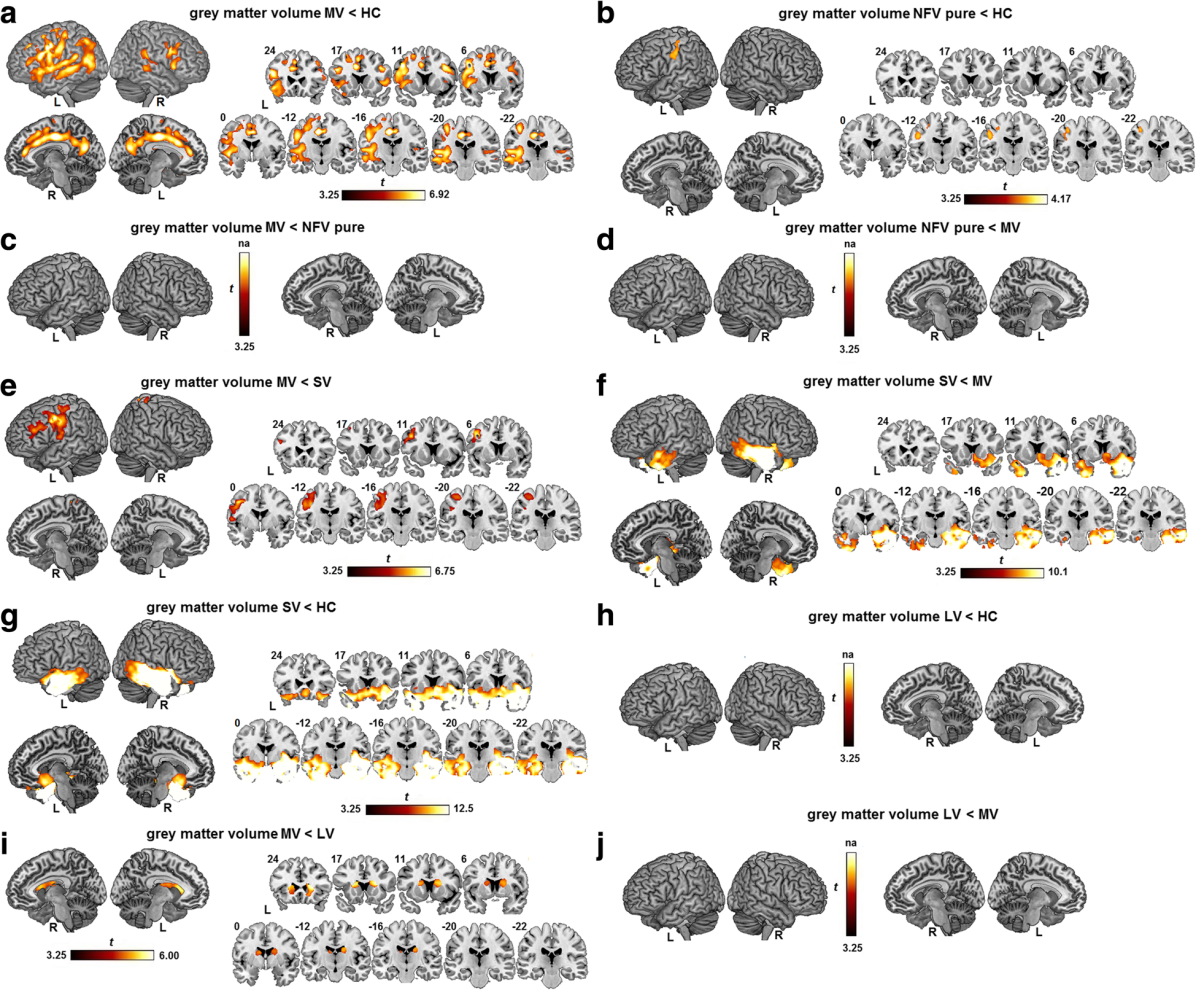
Reduced gray matter volume. Reduced gray matter volume based on a voxel-wise ANOVA with age and gender as covariates, depicted by a one-sided *t* contrast on an MNI template brain rendering and on coronal slices in **a** mixed variant (MV) PPA compared with healthy controls (HC), **b** nonfluent/agrammatic variant (NFV) pure PPA compared with HC, **c** MV compared with NFV pure, **d** NFV pure compared with MV, **e** MV compared with semantic variant (SV), **f** SV compared with MV, **g** SV compared with HC, **h** logopenic variant (LV) compared with HC, **i** MV compared with LV, and **j** LV compared with MV. The significance threshold was set at voxel-level uncorrected *P* < 0.001 with cluster-level family wise error (FWE)-corrected threshold *P* < 0.05. L left, R right

Atrophy in the MV PPA group was relatively widespread and comprised mainly frontal and temporoparietal regions with left-sided predominance (Fig. 3a). The highest degree of atrophy was observed in the premotor cortex, supplementary motor area, pars triangularis and pars opercularis, inferior parietal lobule, insula, precuneus, and in the cingulum bilaterally (Fig. 3a). The superior temporal gyrus and sulcus, left putamen, hippocampus, and perirhinal cortex were also atrophic compared with the healthy control group (Fig. 3a). NFV pure showed atrophy in the left dorsal premotor cortex compared with healthy controls (Fig. 3b). There were no significant differences in gray matter volume between MV and NFV pure at the preset threshold (Fig. 3c, d). MV had significantly more atrophy compared with SV in the left premotor cortex and left inferior frontal sulcus (Fig. 3e). SV in turn had lower gray matter volume in the anterior temporal lobes bilaterally and in the right ventromedial frontal cortex compared with MV (Fig. 3f) and compared with controls (Fig. 3g). MV PPA showed more atrophy in the caudate nuclei bilaterally compared with LV (Fig. 3i). In the current study, LV PPA did not have lower gray matter volume compared with controls (Fig. 3h) or compared with MV (Fig. 3j).

**Fig. 4:**
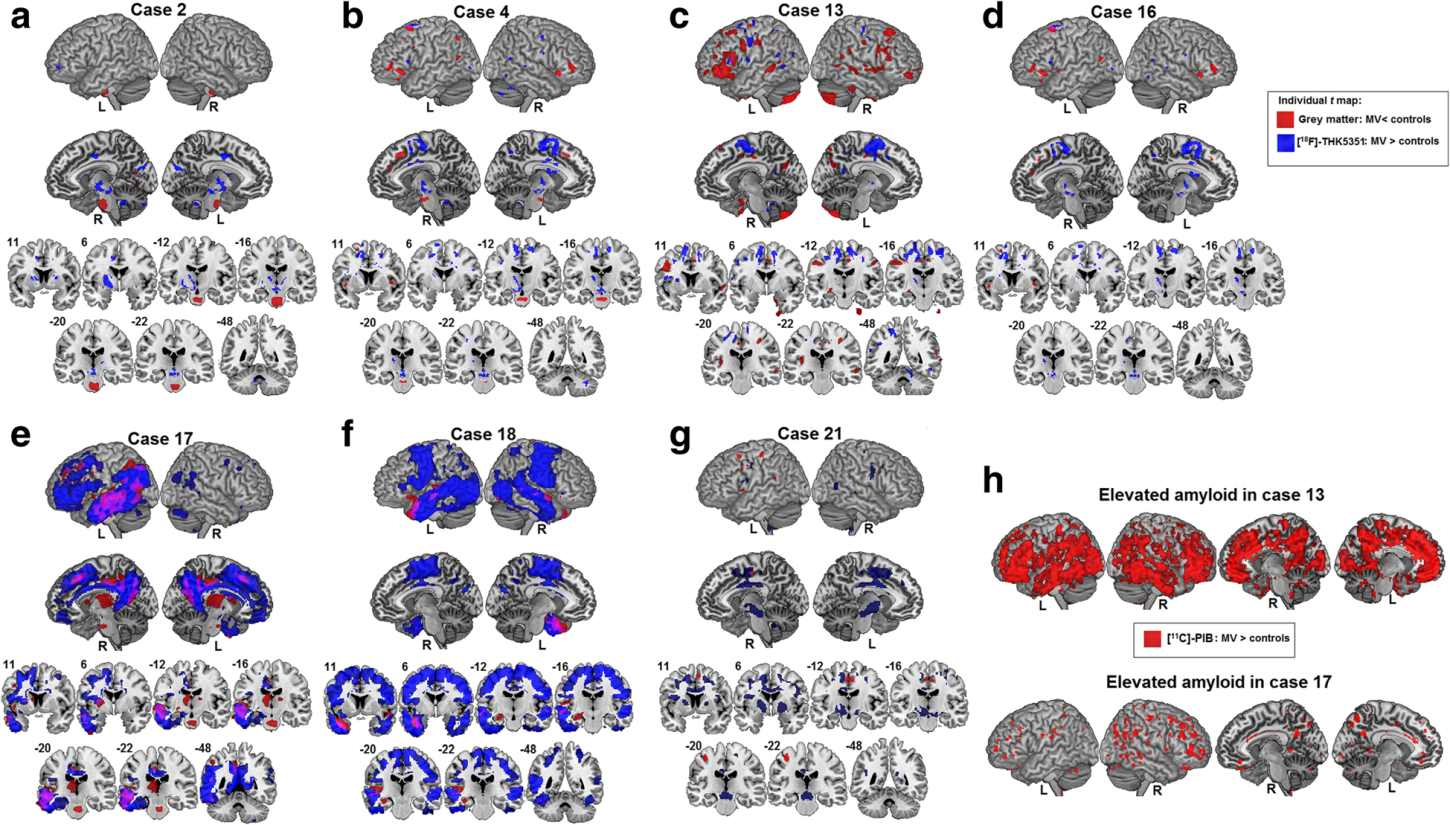
Imaging biomarkers in individual mixed variant (MV) PPA cases. **a–g** Individual *t* maps representing elevated partial volume corrected [^18^F]-THK5351 binding (blue) based on SUVR images of each individual MV PPA case contrasted with 20 healthy controls and reduced gray matter (red) compared with 41 healthy controls. **h** Individual *t* maps of cases 13 and 17 representing elevated amyloid load based on partial volume corrected [^11^C]-Pittsburgh Compound B (PIB) SUVR images contrasted to 14 healthy controls. All individual *t* maps are depicted at a voxel-level uncorrected threshold of *P* < 0.001 contrasting each MV case against a matched group of healthy controls. L left, R right

Individual *T* maps of reduced gray matter volume in MV compared with healthy controls are shown in Fig. 4a–g (shown in red). Atrophy was present in the left inferior frontal gyrus and dorsal premotor cortex in five MV cases (Fig. 4b–e, g; shown in red) and in posterior temporal regions in six MV cases (Fig. 4b–g; shown in red). Three MV cases showed atrophy in the pons (Fig. 4a, b, e; shown in red).

### [^18^F]-THK5351 binding pattern

**Fig. 5:**
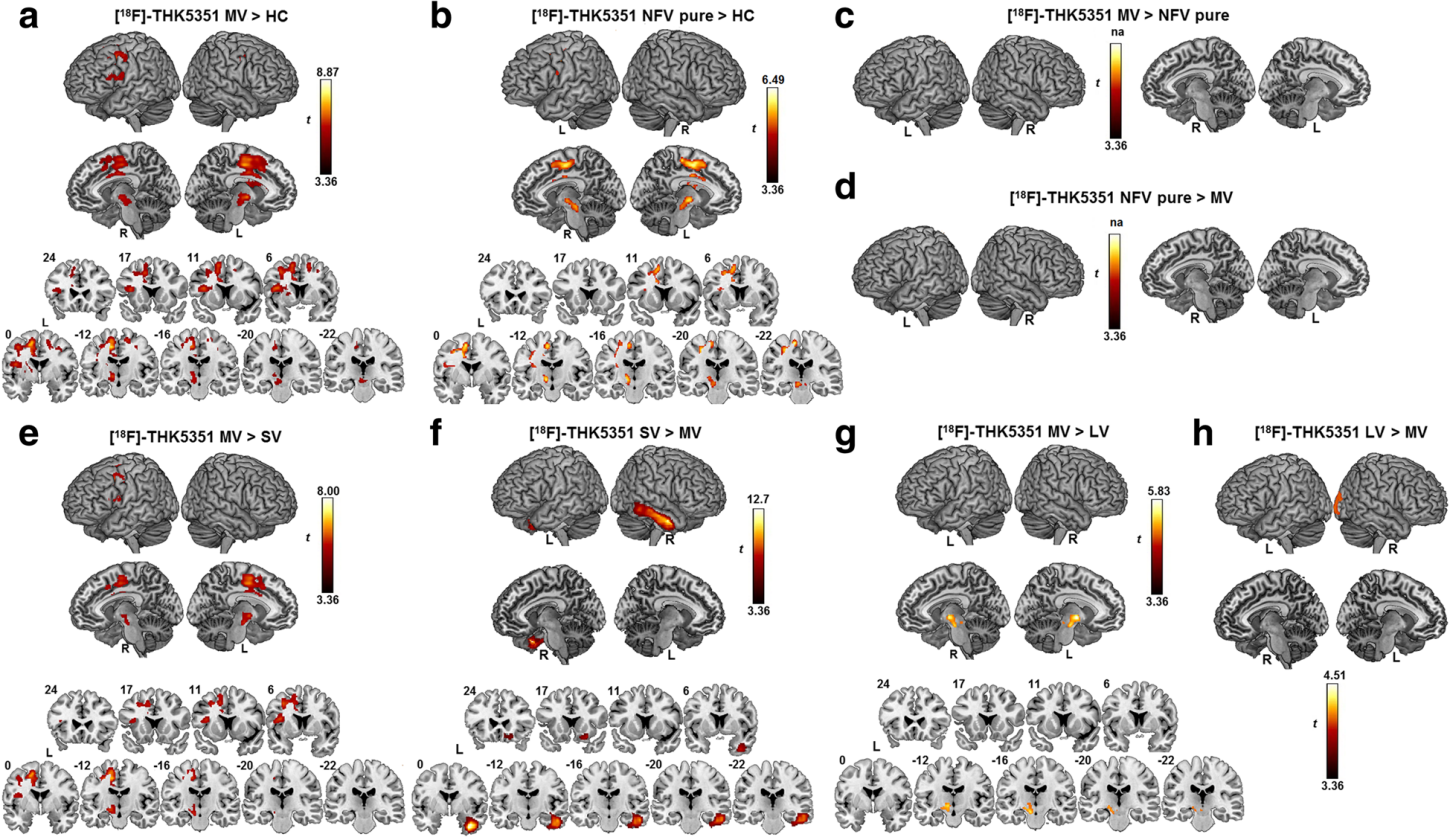
Elevated [^18^F]-THK5351-binding patterns. Elevated [^18^F]-THK5351 binding on partial volume corrected SUVR images, statistically contrasted using a voxel-wise ANOVA with age and gender as covariates, depicted by a one-sided *t* contrast on an MNI template brain rendering and on coronal slices in **a** mixed variant (MV) PPA compared with healthy controls (HC), **b** nonfluent/agrammatic variant (NFV) pure PPA compared with HC, **c** MV compared with NFV pure, **d** NFV pure compared with MV, **e** MV compared with semantic variant (SV), **f** SV compared with MV, **g** MV compared with logopenic variant (LV), and **h** LV compared with MV. The significance threshold was set at voxel-level uncorrected *P* < 0.001 with cluster-level family wise error (FWE)-corrected threshold *P* < 0.05. L left, R right

[^18^F]-THK5351 binding in MV PPA was significantly higher in the supplementary motor area bilaterally and in the left dorsal premotor cortex, left pars triangularis, and pars opercularis extending medially into the insula, basal ganglia, thalamus, subthalamic nucleus, red nucleus, and substantia nigra compared with healthy controls (Fig. 5a). When a more lenient threshold was applied (voxel-level uncorrected *P* < 0.005), additional elevated [^18^F]-THK5351 binding in the superior temporal gyrus and in the lateral temporal lobe was present in MV. An almost identical pattern of [^18^F]-THK5351 binding was present when MV was compared with SV PPA (Fig. 5e). In NFV pure, elevated [^18^F]-THK5351 binding compared with controls was observed in the left supplementary motor area and in the basal ganglia, thalamus, subthalamic nucleus, red nucleus, and substantia nigra (Fig. 5b). MV and NFV pure did not differ in [^18^F]-THK5351 binding at the preset significance threshold (Fig. 5c, d). SV showed elevated [^18^F]-THK5351 binding compared with MV in the right inferior lateral temporal gyrus and in the right ventromedial frontal gyrus (Fig. 5f). MV had increased [^18^F]-THK5351 binding compared with LV in the left substantia nigra, thalamus, and subthalamic nucleus (Fig. 5g), while LV patients had elevated [^18^F]-THK5351 binding compared with MV in the right temporooccipital lobe (Fig. 5h).

Individual *T* maps of elevated [^18^F]-THK5351 binding of all MV PPA patients are shown in Fig. 4a–g (shown in blue). All MV PPA cases showed elevated [^18^F]-THK5351 binding in the medial frontal cortex. More specifically, elevated [^18^F]-THK5351 binding was present in six out of seven cases in the supplementary motor area bilaterally and in the left dorsal premotor cortex (Fig. 4b–g; shown in blue). Five of these six cases showed elevated [^18^F]-THK5351 binding in the thalamus (Fig. 4b–e, g; shown in blue), three in the midbrain including the substantia nigra (Fig. 4b, d, g; shown in blue) and two of these six cases also showed elevated binding in the basal ganglia (Fig. 4e, g; shown in blue). In one case (case 2; Fig. 4a; shown in blue), binding in the basal ganglia, thalamus, cingulum, and midbrain without supplementary motor area/premotor cortical involvement was present. Another case (case 18) showed only elevated [^18^F]-THK5351 binding in cortical regions but not in subcortical regions (Fig. 4f; shown in blue). The latter case, together with case 17 (Fig. 4e; shown in blue) showed extensive [^18^F]-THK5351 binding encompassing almost the entire temporal lobe with a left-hemispheric predominance. This pattern overlapped with the atrophy pattern (red) in these patients (Fig. 4e–f; overlap shown in violet).

### Amyloid biomarker positivity

As a group, MV PPA patients showed significantly elevated [^11^C]-PIB binding in the orbitofrontal cortex, the anterior cingulate cortex, and in the precuneus/posterior cingulate cortex bilaterally compared with healthy controls (Additional file 2: Figure S1). At the individual patient level, two out of seven MV PPA cases (case 13 (76 years old) and case 17 (65 years old)) were amyloid-positive based on either neocortical [^11^C]-PIB PET SUVR values (Fig. 4h) (Table 1) or on CSF Aβ_42_ values (Table 1). MV case 4 and case 16 did not show any elevated [^11^C]-PIB binding, and neither did MV cases 2, 18, and 21 show positivity on CSF Aβ_42_ or CSF Aβ_42_/t-tau (Table 1). In the LV group, all three cases were amyloid-positive (Table 1). In contrast, none of the NFV pure or SV cases were amyloid-positive (Table 1).

## Discussion

This study provides a comprehensive analysis of the prevalence and nature of single-word comprehension problems in PPA, which occurred in a substantial number of cases with speech apraxia and/or agrammatism. These patients were classified as MV PPA and showed additional deficits on object knowledge and object recognition.

We demonstrated for the first time a focal pattern of elevated [^18^F]-THK5351 binding which was narrowly circumscribed and highly similar to NFV pure PPA.

At a clinical level, data-driven mathematical analyses of neurolinguistic and neuropsychological data of PPA patients suggest the existence of a mixed phenotype [25, 26]. In the first study reporting on MV PPA [19], single-word comprehension was assessed by a selected subset of 36 moderately difficult items of the Peabody Picture Vocabulary Test (PPVT) [71]. The proposed cut-off to define cases with MV PPA was a PPVT score and a Northwestern anagram test score < 60% [19]. In the current study, we did not use percentiles to define abnormality of performance but used a regression equation correcting for age or education effects and a modified *t* test assessing each individual case against a healthy control group [52, 64]. To date, no consensus exists, however, on which neuropsychological tests or which cut-offs to apply and thus considerable variation can arise when assigning a diagnosis of PPA. In this series of MV cases, the cut-off for single-word comprehension deficits was a priori defined based on the sum score of the AAT, in which words are presented in the auditory or written modality and need to be matched to a picture [51]. Three MV patients who showed object recognition problems measured with BORB (Table 1) also showed deficits on a purely verbal associative-semantic task (PALPA subtest 49), suggesting that both visual and verbal modalities of semantic representations are affected.

A detailed assessment of the single-word comprehension errors revealed that retrieving the nondominant meaning of a word was particularly problematic in MV PPA. This finding might relate to disturbances in top-down semantic control mechanisms [37, 72, 73]. In SV PPA, word comprehension problems are associated with atrophy of the anterior temporal lobes [29, 74, 75]. This has engendered the hub-and-spoke theory, which considers the anterior temporal pole as a hub region that binds together the distributed representations of the meaning of a word [28, 76]. The mechanism for the single-word comprehension deficits in MV probably differs fundamentally from that postulated in SV and also from the mechanism that may occur in LV. In LV, there is evidence for a phonological short-term memory deficit that may contribute to the hesitancy during spontaneous speech [77, 6]. This is related to atrophy of the temporoparietal junction [77, 6]. The presence of word comprehension deficits in some LV cases may reflect expansion of atrophy to the posterior superior temporal sulcus and might be due to impaired lexical-semantic retrieval [30, 31]. We postulate that the single-word comprehension deficits in MV relate to the role of the inferior frontal cortex in the processing of word meaning [34]. The pars triangularis and the inferior frontal sulcus have been implicated in a variety of semantic processes, including semantic working memory, dynamic uploading of semantic representations, semantic selection, and semantic control [33, 34, 36, 37, 73, 78]. The prominent meaning dominance effect in MV may suggest dysfunctional semantic control processes. Semantic control mechanisms in the pars triangularis would enable one to ignore the distracter picture, which was semantically related to the dominant meaning of that word, and select the less-frequent, nondominant meaning [73]. Meaning dominance effects have previously been located to left middle and superior temporal regions, but also to the right globus pallidus and putamen, based on task-based functional MRI studies in healthy controls [79]. A role of the basal ganglia has been demonstrated in suppressing irrelevant words [80]. Disturbance of frontal-subcortical systems influencing inhibitory semantic mechanisms has been linked to a circumscribed deficit in the selective attentional engagement of the semantic network on the basis of meaning frequency [81]. Meaning dominance effects on single-word comprehension deficits in MV PPA might possibly also relate to damage of white matter tracts connecting the main anterior temporal lobe with regions involved in cognitive semantic control such as the inferior frontal gyrus [34]. This hypothesis remains to be investigated using diffusion-weighted imaging which was not available in the current cohort**.** Deficits in semantic control have also been implied in the word comprehension deficits following left inferior frontal ischemic damage in stroke patients [72, 82]. Further empirical investigation is required to test these hypotheses and determine the origin of the single-word comprehension deficits in MV.

This study was the first to characterize MV PPA with [^18^F]-THK5351 PET. Elevated [^18^F]-THK5351 binding was present bilaterally in the supplementary motor area and left dorsal premotor cortex in both MV and NFV pure PPA (Fig. 5). These regions have been implicated previously in primary progressive apraxia of speech, a syndrome which shows underlying FTLD tauopathy at postmortem examination [12] and elevated retention of the tau PET tracer [^18^F]-AV1451 in the supplementary motor area, dorsal premotor cortex, and inferior frontal gyrus [11]. The supplementary motor area plays a crucial role in speech motor control [83], and premotor cortical involvement has been linked with the severity of speech apraxia [84]. Damage to the white matter tract connecting the supplementary motor area with the inferior frontal gyrus (i.e., the aslant tract) [16, 85, 86] affects the amount of distortion errors that NFV PPA patients make in spontaneous speech [86]. Apraxia of speech features in the current series of MV cases would be categorized as the ‘phonetic type’, dominated by sound distortions and distorted sound substitutions [12–14, 87, 88]. While apraxia of speech is not included in the proposed diagnostic criteria for MV PPA [20], all MV PPA cases in a previous case series also showed apraxia of speech accompanied by agrammatism and single-word comprehension deficits [27]. In that study, atrophy of the premotor cortex was observed in MV PPA, which could possibly be linked with their features of speech apraxia. We noticed that, by applying a more lenient statistical threshold, [^18^F]-THK5351 binding was also present in temporal regions in MV PPA but not in NFV PPA. The temporal lobe was also affected by atrophy in MV, which was consistent with previous MRI-based findings [20, 27]. Loss of the structural integrity of posterior temporal regions is associated with single word-comprehension problems [28, 30] and with prominent agrammatic features [84]. The left inferior frontal gyrus and middle frontal gyrus showed atrophy both in MV and in NFV PPA. These regions have been implicated in sentence comprehension as an index for agrammatism [89, 90].

Elevated [^18^F]-THK5351 binding in MV PPA did not only encompass cortical regions but also involved subcortical regions; i.e., the midbrain, thalamus, and basal ganglia. These regions are typically vulnerable to FTLD tauopathy corresponding to corticobasal degeneration (CBD) or progressive supranuclear palsy (PSP) pathology [91, 92], which can be visualized with the [^18^F]-THK5351 tracer [93, 94]. The striatum and subthalamic nucleus are involved early in the disease course of CBD, while the substantia nigra can be involved in later stages of the disease [92, 95]. A subset of MV and NFV cases in this study showed mild clinical signs and symptoms that may be indicative of underlying PSP or CBD pathology, including right-sided extrapyramidal signs or vertical eye movement abnormalities (Table 2). While the initial and most salient feature in these MV PPA cases was the language and speech impairment, these patients may develop a PSP- or CBD-like syndrome over time, similar to what has been reported to occur in patients with primary progressive apraxia of speech [42].

Besides binding to FTLD tauopathy, [^18^F]-THK5351 also binds to tau pathology of the Alzheimer’s disease type [38]. At the individual patient level, the two amyloid-positive MV PPA cases showed elevated [^18^F]-THK5351 binding in the left temporoparietal junction but not in the midbrain (Fig. 4). This suggests that these MV cases have underlying Alzheimer’s disease pathology. This would be in line with the positive amyloid PET result, indicative of an increased fibrillary amyloid load [39]. Amyloid PET positivity should, however, be cautiously interpreted in this age group, as it may also occur in the absence of cognitive deficits [43, 48, 63, 96]. However, numerically a proportion of two out of seven (29%) amyloid PET-positive cases exceeds the expected proportion based on studies in healthy controls of similar age (15%) [43, 48, 63]. The proportion of amyloid-positive MV cases in this study is lower than observed in a previous PPA study, which demonstrated amyloid-positivity in three out of four MV cases based on amyloid PET [10]. The latter study also demonstrated a higher prevalence of amyloid-positivity in MV compared with NFV pure cases [10]. This is consistent with our findings, but obviously must be considered preliminary given the low sample size. There were no differences in age between MV and NFV pure PPA and therefore age is unlikely to account for the higher prevalence of amyloid positivity in the MV cases. In one postmortem study, Alzheimer’s disease pathology was present in four out of six MV cases [7]. In another series, the prevalence of Alzheimer’s disease pathology was lower in MV PPA (25%, one out of four) and FTLD tauopathy was more prevalent (75%, three out of four) [27]. However, the negative amyloid PET in most MV patients virtually rules out Alzheimer’s disease as the underlying cause of the cognitive deficits.

### Implications for PPA classification

The current findings may have implications for possible revisions of the currently recommended PPA classification scheme [1]. In summary, the two most relevant points taken from the current study are the following. First, speech apraxia and agrammatism were relatively commonly associated with single-word comprehension deficits. In these patients, object knowledge was also mildly deficient according to standard neuropsychological tests. These deficits were, however, less pronounced than those seen in SV. Second, the underlying neurobiology did not appear fundamentally different between NFV pure and MV; [^18^F]-THK5351 binding patterns were comparable between MV and NFV pure, and structural MRI did not reveal a significant difference. Taken together, these two main observations do not justify the addition of a fourth subtype to the PPA classification scheme, principally given the close neurobiological similarity between MV and NFV pure as testified by the similarity in [^18^F]-THK5351 binding patterns. On the other hand, the exclusionary criteria of spared single-word comprehension and object knowledge for NFV might be questioned based on the current findings. Making these two exclusionary criteria less restrictive would provide for a proper classification of the cases we described as MV within the current three-variants scheme [1].

### Study limitations

No firm conclusions regarding the underlying type of tauopathy can be drawn as this study is limited by the lack of postmortem confirmation. Furthermore, the total number of PPA patients included is rather small, which is partly inherent to the relatively low prevalence of the syndrome. A drawback of using [^18^F]-THK5351 PET is the nonspecific binding to an undefined molecular substrate in the basal ganglia [38, 40]. In-vivo experiments using selegiline displacement in patients with Alzheimer’s disease and PSP indicate that [^18^F]-THK5351 may bind to MAO-B [40], suggesting that [^18^F]-THK5351 binds to astrogliosis. Nonetheless, elevated [^18^F]-THK5351 binding in the current study was highly focalized and colocalized with regions known to be associated with conditions in which a tauopathy is the underlying neuropathological cause [91, 92, 97, 98].

## Conclusions

A PPA subtype characterized by speech apraxia and/or agrammatism with concomitant single-word comprehension problems in an early disease stage clearly exists. MV PPA showed focal [^18^F]-THK5351 PET binding in the supplementary motor area, premotor cortex, midbrain, and basal ganglia, highly similar to NFV pure. Given the high neurobiological similarity, the addition of a fourth subtype to the three currently used subtypes is not warranted based on the current data. However, the exclusionary criteria of spared single-word comprehension and object knowledge for NFV may need to be reconsidered based on the current data. At a basic scientific level, the relatively frequent occurrence of single-word comprehension problems in NFV resonates with the increasing evidence for a role of the inferior frontal cortex in a variety of semantic processes.

## Additional files

Additional file 1: **Table S1.**Demographics of cognitively intact older control groups. Abbreviations: [^11^C]-PIB [^11^C]-Pittsburgh Compound B, MRI magnetic resonance imaging, PPA primary progressive aphasia. (DOCX 13 kb)

Additional file 2: **Figure S1.**Elevated amyloid load was measured on [^11^C]-Pittsburgh Compound B ([^11^C]-PIB) SUVR images contrasted using a voxelwise ANOVA with age and gender as covariates, depicted by a one-sided t contrast on an MNI template brain rendering and on coronal slices in (A) mixed variant (MV) PPA compared to healthy controls (HC), (B) MV compared to semantic variant (SV) PPA, (C) logopenic variant (LV) compared to MV. The significance threshold was set at voxel-level uncorrected *P* < 0.001 with cluster-level family wise error (FWE)-corrected threshold *P* < 0.05. L left, R right. (TIF 1257 kb)

## Abbreviations

[^11^C]-PIB: [^11^C]-Pittsburgh Compound B
AAT: Aachen Aphasia Test
ANOVA: Analysis of variance
AVF: Animal Verbal Fluency
Aβ_1–42_: Amyloid-β_1–42_
BNT: Boston Naming Test
BORB: Birmingham Object Recognition Battery
CBD: Corticobasal degeneration
CDR: Clinical Dementia Rating
CPM: Colored Progressive Matrices
CSF: Cerebrospinal fluid
CT: Computed tomography
DIAS: Diagnostisch Instrument voor Apraxie van de Spraak
FTLD: Frontotemporal lobar degeneration
FWE: Family wise error
FWHM: Full-width half-maximum
LV: Logopenic variant
MAO-B: Monoamine oxidase-B
MMSE: Mini-Mental State Examination
MRI: Magnetic resonance imaging
MV: Mixed variant
NFV: Nonfluent/agrammatic variant
p_181_-tau: Phospho_181_-tau
PALPA: Psycholinguistic Assessment of Language Processing in Aphasia
PET: Positron emission tomography
PPA: Primary progressive aphasia
PPT: Pyramids and Palm trees Test
PPVT: Peabody Picture Vocabulary Test
PSP: Progressive supranuclear palsy
PVC: Partial volume corrected
SUVR: Standardized uptake value ratio
SV: Semantic variant
t-tau: Total tau
WEZT: Werkwoorden En Zinnen Test
