# Supplementary material for: Single-word comprehension deficits in the nonfluent variant of primary progressive aphasia
Source: Alzheimers Res Ther. 2018 Jul 18;10:68. doi: 10.1186/s13195-018-0393-8 (PMC6052568; doi:10.1186/s13195-018-0393-8)
Supplement: Supplementary file 1 — Table S1.Demographics of cognitively intact older control groups. Abbreviations: [11C]-PIB [11C]-Pittsburgh Compound B, MRI magnetic resonance imaging, PPA primary progressive aphasia. (DOCX 13 kb) [file 13195_2018_393_MOESM1_ESM.docx]

| **Healthy control group** | **N** | **Gender (male/female)** | **Comparison to PPA *(P* value*)*** | **Median age (range)(years)** | **Comparison to PPA *(P* value*)*** | **Median education (range)(years)** | **Comparison to PPA *(P* value*)*** |
| --- | --- | --- | --- | --- | --- | --- | --- |
| Neuropsychology | 64 | 30/34 | 0.68 | 67.5 (53-89) | 0.50 | 13.0 (8-22) | 0.76 |
| [^18^F]-THK5351 | 20 | 10/10 | 0.75 | 69.5 (58-80) | 0.48 | 13.5 (8-20) | 0.23 |
| MRI | 41 | 20/21 | 0.78 | 70 (58-81) | 0.16 | 13.0 (8-20) | 0.28 |
| [^11^C]-PIB | 33 | 21/12 | 0.44 | 73.0 (59-80) | <0.001 | 13.0 (8-20) | 0.73 |

**Supplementary Table 1. Demographics of cognitively intact older control groups**
