# Supplementary figures and images for: Single-word comprehension deficits in the nonfluent variant of primary progressive aphasia
Source: Alzheimers Res Ther. 2018 Jul 18;10:68. doi: 10.1186/s13195-018-0393-8 (PMC6052568; doi:10.1186/s13195-018-0393-8)

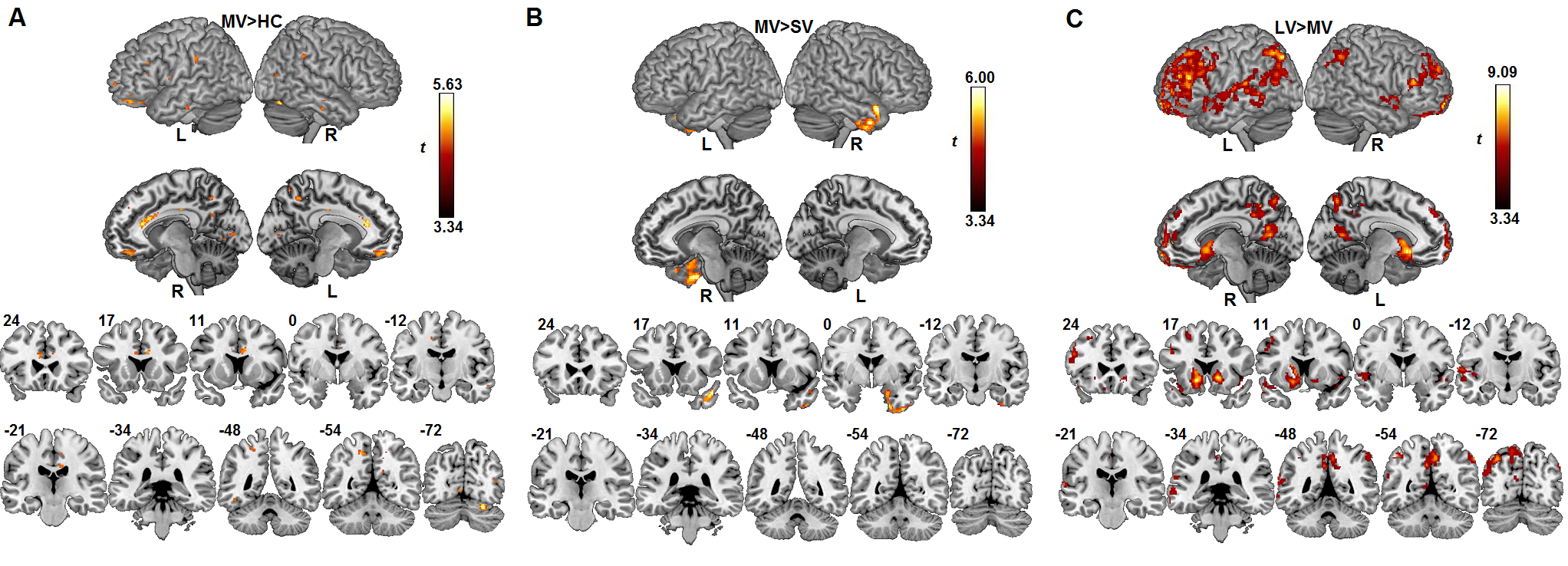

Supplement: Supplementary file 2 — Figure S1.Elevated amyloid load was measured on [11C]-Pittsburgh Compound B ([11C]-PIB) SUVR images contrasted using a voxelwise ANOVA with age and gender as covariates, depicted by a one-sided t contrast on an MNI template brain rendering and on coronal slices in (A) mixed variant (MV) PPA compared to healthy controls (HC), (B) MV compared to semantic variant (SV) PPA, (C) logopenic variant (LV) compared to MV. The significance threshold was set at voxel-level uncorrected P < 0.001 with cluster-level family wise error (FWE)-corrected threshold P < 0.05. L left, R right. (TIF 1257 kb) [file 13195_2018_393_MOESM2_ESM.tif]
